# 870. Frostbite Within Homeless Population: A Retrospective Analysis

**DOI:** 10.1093/jbcr/irag033.347

**Published:** 2026-04-06

**Authors:** Eric Sleasman, Kiernan Gunn, Siddharth Chittaranjan, Piper Schneider, Samantha Shellen, Keith R Sweitzer, Brielle Raine, Derek Bell

**Affiliations:** University of Rochester Medical Center, Rochester, NY; University of Rochester Medical Center, Rochester, NY; University of Rochester Medical Center, Rochester, NY; University of Rochester Medical Center, Rochester, NY; University of Rochester Medical Center, Rochester, NY; University of Rochester Medical Center, Rochester, NY; University of Rochester Medical Center, Rochester, NY; University of Rochester Medical Center, Rochester, NY

## Abstract

**Introduction:**

Frostbite is a limb-threatening injury that requires rapid treatment to reduce significant morbidity. Homeless populations have been described to be disproportionately affected by frostbite injuries due to socioeconomic challenges. Few studies have evaluated modifiable delays within the acute care model of treatment that directly influence homeless person care.

**Methods:**

This study used retrospective review to analyze frostbite admissions at a single, ABA verified Burn Center from 2015 and 2025. Patients were placed into non-homeless (n = 202) and homeless (n = 31) groups. Admission-to-consultation time (ATC) and length of hospital stay (LOS) were analyzed after excluding zero wait times from the non-homeless group. Frostbite severity was calculated using total body surface area (TBSA) of combined 2nd and 3rd degree injuries. Operating room (OR) visits, number of OR visits, and procedure type were also evaluated. Data was not normally distributed by Shapiro Wilks test, so Mann–Whitney U Test was used for comparisons. R was used to evaluate correlation between ATC and LOS and Fisher’s exact test was used to compare surgery type.

**Results:**

ATC was longer in homeless patients (median 4.1 h [IQR 2.6–16.4]) vs non-homeless (median 2.99 h [1.44–5.45], *p*=.02). LOS was also greater for homeless (median 6d [IQR 3–22]) vs non-homeless (median 1d [IQR 1–7.25], *p*<.0001). ATC and LOS showed weak correlation (r = 0.136). Frostbite severity (TBSA of 2nd/3rd degree burns) was similar for homeless (median TBSA 0.8 [IQR 0.38–3.55]) vs non-homeless (median 0.57 TBSA [IQR 0.16–1.49], *p*=.06). OR visits for homeless (median 3 visits [IQR 0–4]) vs non-homeless (median 3 visits [IQR 0–3], *p*=.70), time to OR for homeless (median 6d [IQR 1–8]) vs non-homeless (median 1.5d [IQR 0–8.25], *p*=.16), surgery rates (*p*=.32), and surgery type (salvage vs. amputation (*p*=.75)) did not differ.

**Conclusions:**

Homeless patients with frostbite experienced significantly longer ATC times and prolonged LOS compared to non-homeless patients despite similar frostbite severity and treatments. As both patient groups show similar surgical rates, OR visits, surgery types, and disease severity, increased ATC and LOS are likely driven by social factors rather than treatment. Furthermore, as weak correlation suggests LOS is not dependent on ATC, we conclude that ATC and LOS may be two fundamentally, different challenges in need of separate investigations.

**Applicability of Research to Practice:**

This study identifies correctable pitfalls in frostbite management for homeless patients and shows that once care is initiated, patients receive comparable surgical treatment. This study highlights care disparities within ATC and LOS as two targets for improving frostbite care in the homeless population. System and social improvements such as consult protocols, frostbite education for triage personnel, or more efficient discharge pathways may reduce cost, leading to more equitable care and reduced morbidity.

**Funding for the study:**

N/A.

